# Mining the Gut Microbiota for Microbial-Based Therapeutic Strategies in Cancer Immunotherapy

**DOI:** 10.3389/fonc.2021.721249

**Published:** 2021-09-13

**Authors:** Bolei Li, Tao Gong, Yu Hao, Xuedong Zhou, Lei Cheng

**Affiliations:** ^1^State Key Laboratory of Oral Diseases, West China Hospital of Stomatology, National Clinical Research Center for Oral Diseases, Sichuan University, Chengdu, China; ^2^Department of Operative Dentistry and Endodontics, West China School of Stomatology, Sichuan University, Chengdu, China

**Keywords:** gut microbiota, cancer immunotherapy, MAMPs, microbial metabolites, fecal microbiota transplant

## Abstract

The past two decades witnessed a revolution in our understanding of host–microbiota interactions that led to the concept of the super-organism consisting of a eukaryotic part and a prokaryotic part. Owing to the critical role of gut microbiota in modulating the host immune system, it is not beyond all expectations that more and more evidence indicated that the shift of gut microbiota influenced responses to numerous forms of cancer immunotherapy. Therapy targeting gut microbiota is becoming a promising strategy to improve cancer immunotherapy. In this review, we discuss the role of the gut microbiota in response to cancer immunotherapy, the mechanisms that the gut microbiota influences cancer immunotherapy, and therapeutic strategies targeting gut microbiota to improve cancer immunotherapy.

## 1 Introduction

Over the past decades, immunotherapy has emerged as a mainstay in cancer treatment, with the advances in our understanding of cancer immunosuppressive microenvironments. Cancer immunotherapy was applied to a broad range of cancers, but 70% to 80% of patients failed to experience a life-altering durable response (1). To benefit more patients from cancer immunotherapy, efforts are made to evoke the immune response.

The gut microbiota is drawing tremendous attention given its effects on human health. Mounting evidence revealed that the gut microbiota and the immune system constantly interact (2, 3). Since immunotherapy was approved by US Food and Drug Administration (FDA), increasing clinical studies revealed the association between the gut microbiota and response to immunotherapy. Basing on the solid clinical association, the causal/mechanistic link of gut microbiota and immunotherapy was uncovered with preclinical models. Microbe-associated molecular patterns (MAMPs), molecular mimicry of microbial antigens with tumor neoantigen, and microbial metabolites were key factors that gut microbiota depends on to influence the response of cancer immunotherapy. Currently, more and more preclinical and clinical evidence indicated that the shift of gut microbiota influenced responses to numerous forms of cancer immunotherapy (4). As a result, therapeutic strategies targeting gut microbiota, including fecal microbiota transplant (FMT), diet, probiotics, and antibiotics, are regarded as promising candidates in improving cancer immunotherapies. Numerous clinical trials were performed to explore effective strategies to benefit cancer immunotherapy *via* improving gut microbiota. Thus, this review will mine the gut microbiota for cancer immunotherapy *via* summarizing and discussing the clinical-associated and causal/mechanistic links and clinical trials of gut microbiota and cancer immunotherapy, comparing the advantages and disadvantages of therapeutic strategies targeting the gut microbiota.

### 1.1 Cancer Immunotherapy

The immune system plays a dominant role in cancer control, attributed to the detection and elimination of cancer cells. On the other hand, some tumor cells escape immune surveillance by i) defecting the expression of antigen-presenting proteins, or antigen processing, or presentation, rendering them invisible to immune cell; ii) expressing proteins in inhibiting inflammation and inducing an immunosuppressive state within the tumor microenvironment; and iii) becoming insensitive to immune effector mechanisms (5). Immunotherapy helps the immune system to better act against cancer, *via* encouraging immune elimination and hindering immune evasion of cancer cells.

Therapeutic advances in immunotherapy have rapidly emerged in the past few years, especially the immune checkpoint inhibitors (ICIs). Currently, ICIs are FDA-approved for the treatment of many cancer types, including advanced-stage melanoma, squamous and non-squamous non-small cell lung carcinoma (NSCLC), Merkel cell carcinoma, head and neck squamous cell carcinoma, urothelial carcinoma, kidney carcinoma, microsatellite instability-high or DNA mismatch repair-deficient cancers, refractory Hodgkin lymphoma, hepatocellular carcinoma, and gastric cancer (6, 7). Now, ICIs are coming to neoadjuvant (presurgical) era. Clinical studies (8–10) have unleashed the promise of neoadjuvant immunotherapy. More than 90% of NSCLC patients were able to undergo surgery within the planned timeframe after neoadjuvant immunotherapy (11). In addition, RNA vaccine could be another effective immunotherapy, which drives immunity by the induction of strong CD4^+^ and CD8^+^ T-cell immunity against the vaccine antigens to kill cancer cells (12).

Despite the successful application of cancer immunotherapy across a broad range of human cancers, only 20% to 30% of patients experience life-altering durable response from these therapies, which varies depending on the tumor type (1). Indeed, immunotherapy responses are heterogeneous; most patients manifest primary or secondary resistance to ICIs or even acceleration of the disease, which is called “hyperprogression” (13). Efforts are being made to identify the parameters that govern the threshold of the immunity to evoke the effective anticancer immune response, defined as the “cancer immune set-point” (14).

Numerous factors have been identified to contribute to the “cancer immune set-point” *via* regulating overall immune status, including tumor mutational load, cell metabolism, genomic drivers, and host-specific genetic variation (15, 16). Also, recent investigations highlight the effect of microbiota on the parameters that govern the effectiveness of immunotherapy (17, 18).

### 1.2 Gut Microbiota and Immunity

The human gastrointestinal tract harbors extremely high densities of microorganisms called the microbiota. A human being is more and more perceived as a super-organism consisting of a eukaryotic part and a prokaryotic part (19, 20). The gut microbiota is populated with as many as 100 trillion cells (21), whose collective gene set is approximately 100 times larger than the human gene complement (22, 23). Since birth, gut microbiota interacts with the host constantly throughout development. In consequence, it is not beyond all expectations that gut microbiota plays an important role in numerous host functions including immunity (2, 17).

In addition to influencing localized immune responses, what is more, gut microbiota contributes to systemic innate and adaptive immunity. On the one hand, the gut microbiota is a main source of MAMPs and ligands of pattern recognition receptors (PRRs). PRRs include the Toll-like receptors (TLRs), the nucleotide-binding oligomerization (NOD)-like receptors (NLRs), the RIG-I-like receptors, the C-type lectin receptors, the absent in melanoma 2 (AIM2)-like receptors, and the OAS-like receptors (24), which are widely expressed innate immune cells. In addition, gut microbiota stimulates the expression of PRRs. For example, gut microbiota orchestrates TLR expression on intestinal epithelial cells (25). MAMPs systemically prime the innate immune system, enhancing killing by bone marrow-derived neutrophils (26, 27) and increasing constitutive production of type I interferons of plasmacytoid dendritic cells (DCs) and cross-priming of DCs (28, 29).

On the other hand, gut microbiota-derived metabolites educate both innate and adaptive immunity. The gut microbiota metabolized the fiber, subsequently increasing the concentration of circulating short-chain fatty acids (SCFAs). SCFAs enhance the generation of macrophage and DC precursors and their phagocytic capacity (30), induce anti-inflammatory regulatory T cells (Tregs) (31), and facilitate antibody production of B cells (32). Polysaccharide A (PSA), a zwitterionic capsular carbohydrate, induces FOXP3^+^ Treg differentiation and the production of IL-10 (33). Purine metabolite inosine advances Th1 differentiation *via* adenosine 2A receptors (A_2A_R) (34). Therefore, it is not surprising that more and more studies are revealing the associations and mechanisms between gut microbiota and cancer immunotherapy and are exploring the strategies to improve immunotherapy by taking advantage of gut microbiota.

### 1.3 The Mechanisms of Gut Microbiota Modulating Immunotherapy

**Figure 1:**
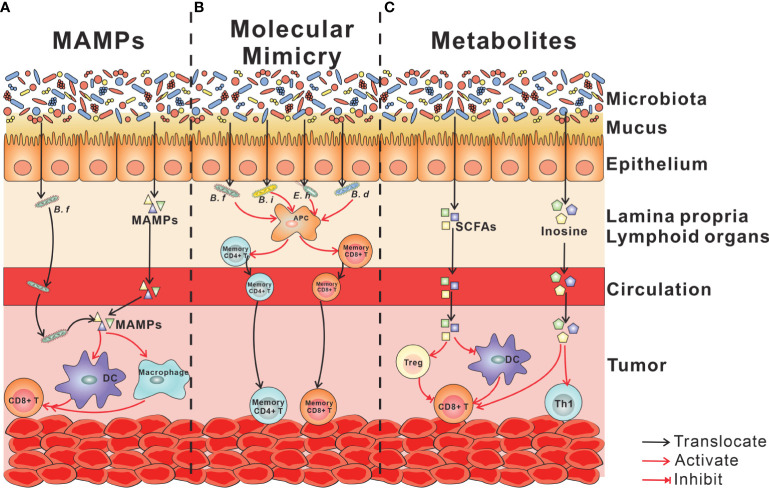
Mechanisms linking gut microbiota with cancer immunotherapy. **(A)** MAMPs. Live bacteria (*Bifidobacterium facilitates*) and MAMPs traverse the mucosal barrier, enter the circulation, and finally locate at the tumor tissue, where MAMPs activate myeloid cells, including DCs and macrophages. The activation of myeloid cells enhances the phagocytosis of macrophages and cytotoxicity of CD8^+^ T cells downstream. **(B)** Molecular mimicry of microbial antigens with tumor neoantigen. Antigens of commensal bacteria, including *Bifidobacterium facilitate*, *Bifidobacterium intestinihominis*, *Enterococcus hirae* 13144, and *Bifidobacterium breve*, are presented by APCs to CD4^+^ T cells and CD8^+^ T cells. By circulations, antigen-specific T cells arrive at tumor tissue and cross-react with tumor neoantigen. **(C)** Microbial metabolites. Microbiota-derived SCFAs play an immune-suppressive role in the tumor microenvironment *via* increasing the portion of Tregs, inhibiting DC maturation and CD8^+^ T-cell activation. Microbiota-derived inosine acts to advance Th1 differentiation and CD8^+^ T cytotoxicity. MAMPs, microbe-associated molecular patterns; DC, dendritic cell; APCs, antigen-presenting cells; SCFAs, short-chain fatty acids.

Diverse studies revealed that gut microbiota plays a crucial part in cancer immunotherapy. Both of the bacteria colonized in the gut and that translocated in the tumor or lymphoid organs regulate cancer immunotherapy. The mechanism for the immune modulation of gut microbiota is being disclosed. Based on existing researches, there are three ways by which gut microbiota influence systemic cancer immunotherapy: a) evoking the innate immunity and downstream adaptive immunity by MAMPs; b) yielding an endogenous tumor vaccine by molecular mimicry of microbial antigens with tumor neoantigen; and c) stimulating tumor-infiltrating immune cell by microbial metabolites (**Figure 1**).

#### 1.3.1 Microbe-Associated Molecular Patterns

MAMPs, ligands of PRRs mostly expressed on innate immune cells, can act directly on local intestinal tissue cells but also penetrate beyond the mucosa, into circulation to tune immune cells in peripheral tissues (35). MAMPS can trigger at least partial activation of innate immune cells such as DCs. Furthermore, conditional antigen-presenting cells (APCs) enhanced the ability to evoke adaptive immune response and modulates cancer immunotherapy (**Figure 1A**).

Commensal bacteria have been identified in extragastrointestinal tissues typically considered to be sterile. Bacteria were detected in the blood (36), lymphoid organs (37, 38), and various tumor tissues (39, 40). Live bacteria gaining access to tumors or lymphoid organs may initiate a strong immune response by MAMPs. For example, the stimulator of interferon genes (STING) is a direct sensor of bacterial cyclic dinucleotides. Shi et al. revealed that *Bifidobacterium* facilitates translocation in tumor sites, where it facilitated anti-CD47 immunotherapy *via* STING signaling, increasing cross-priming of DCs (28) (**Figure 1A**). Sivan et al. showed that splenic DCs isolated from mice colonized with *Bifidobacterium* showed superior priming of naïve CD8^+^ T cells *in vitro* (41).

MAMPs can traverse the mucosal barrier and enter the circulation. Stimuli capable of activating a range of TLR and NOD receptors were detected in serum from healthy individuals (42). In cancer immunotherapy, gut microbiota enhanced cancer response to the combination of CpG and anti-IL-10R through increasing tumor necrosis factor (TNF) production, which depends on the activation of TLR4 on tumor amyloid cells. And gavage with bacterial lipopolysaccharide (LPS), a ligand of TLR4, largely restored TNF production in tumors of antibiotic-treated mice (43). In addition, the activation of macrophages by MAMPs enhanced the phagocytic capability (44) and then primed CD8^+^ T cells to exhibit cytotoxic function (45) (**Figure 1A**).

#### 1.3.2 Molecular Mimicry of Microbial Antigens With Tumor Neoantigen

The theory of “molecular mimicry” posits that T cells elicited by bacteria or viruses accidentally recognize autoantigens as they “escape” from self-tolerance-inducing mechanisms. There were some reports that had demonstrated that microbe-specific CD4^+^ or CD8^+^ T lymphocytes attack normal tissues (46–48). Some data revealed a mechanistic role for T-cell epitopes shared between bacteria and tumor cells (37, 49–53). Fluckiger et al. (53) found the MHC-I-binding epitopes in the tail length tape measure protein (TMP) of a prophage. *Enterococcus hirae* 13144 harbored the bacteriophage that improves the response to anti-PD1 *via* activating TMP-specific H-2Kb-restricted CD8^+^ T cell. In mouse models, administration of enterococci containing the bacteriophage boosted T-cell responses. In humans, the presence of the bacteriophage was associated with improved survival after PD-1 immunotherapy. In addition, *E. hirae* and *Bifidobacterium intestinihominis* specific memory CD4^+^ T cells were associated with longer progression-free survival (PFS) in cancer patients (37). Memory T-cell responses against *Bifidobacterium fragilis* and anticancer efficacy of anti-CTLA4. Adoptive transfer of *Bi. fragilis*-reactive CD4^+^ T cells restored anti-CTLA4 efficacy in germ-free (GF) mice (51). Bessell et al. (52) found that T cells targeting an epitope called SVYRYYGL, expressed in *Bifidobacterium breve*, cross-react with a model neoantigen SIYRYYGL. Compared with mice with *Bifidobacterium* colonization, tumors expressing the model SIYRYYGL neoantigen grew faster in mice lacking *Bifidobacterium* (**Figure 1B**).

#### 1.3.3 Microbial Metabolites

Microbiota can metabolize dietary components that cannot be metabolized by the host, thus contributing to the production of primary metabolites and the modulation of secondary metabolites (54). The diverse array of metabolites in the mammalian intestine have the potential to modulate immunity. Several such microbial metabolites include SCFAs, lactic acid, spermidine, niacin, indole, retinoic acid, PSA, bile acid, and taurine (55).

SCFAs, namely, acetate, propionate, and butyrate, are the result of non-digestible carbohydrate fermentation by anaerobic commensal bacteria. In terms of immune regulation, SCFAs modulate cytokine releasing (56–58) and function of innate immune cells (30, 59, 60), B cell (61), and Tregs (31, 62) by acting as a histone deacetylase inhibitor or ligands for G-protein-coupled receptors. In cancer immunotherapy, SCFAs play an immune-suppressive role with an increase in the abundance of Tregs (63). In the mouse model, administration of butyrate diminished the efficacy of anti-CTLA4, *via* inhibiting DC maturation and T-cell activation (**Figure 1C**). In the clinical study, cancer patients with low concentrations of SCFAs showed prolonged PFS, and an association between gut bacteria and systemic concentrations of SCFAs was found (63). However, these results are in contrast those of with two clinical studies showing that high concentrations of fecal and plasma SCFAs were associated with a response to PD-1 treatment (64, 65).

Although there is no evidence showing that lactic acid or spermidine from gut microbiota influence immunotherapy directly, lactic acid derived from cancer cells suppressed the function of T cells, NK cells, and macrophages, resulting in the attenuated efficiency of anti-PD-L1 and anti-CD47 (66, 67). Gut microbiota-derived spermidine preferentially induces naïve T cells to Tregs in the gut tissue (68).

The purine nucleoside inosine is generated by deamination of adenosine or the action of 5′-nucleotidase on inosine monophosphate. He et al. (69) revealed that gut microbiota regulated levels of the purine metabolite inosine that suppressed the differentiation and inflammation of Th1/Th2 cells *via* A_2A_R on T cells. Intriguingly, Mager et al. (34) discovered that the inhibition of Th1/Th2 cells is dependent on the absence of IFNγ; when this cytokine is present, inosine acted to advance Th1 differentiation *via* A_2A_R and boost anti-CTLA4 therapy. In addition, the translocation of inosine-producing bacteria in tumors was not required for the enhancement of immune therapy. Thus, microbiota-derived soluble inosine augments cancer immunotherapy through blood circulation. Besides signaling molecules, inosine is an essential cellular energy. Within tumors, cancer cells rapidly deplete glucose such that infiltrating T cells, which require abundant energy substrates for full function, would have been outcompeted if alternative substrates were not present. Wang et al. (70) demonstrated that inosine is an alternative source of energy to glucose within the tumor microenvironment; the combination of inosine supplementation and administration of anti-PD-L1 led to delayed tumor growth and increased survival time in a mouse model of melanoma. Unfortunately, some cancer cells compete with T cells for inosine as an energy source, which diminished the beneficial effect of inosine supplementation together with anti-PD-L1 (**Figure 1C**).

## 2 Gut Microbiota in Response and Toxicity to Immunotherapy

### 2.1 Gut Microbiota and Immunotherapy

#### 2.1.1 Clinical Evidence Linking Gut Microbiota and Immunotherapy

Several clinical studies, involving Americans, Chinese, Japanese, French, and Netherlands, have demonstrated the association between gut microbiota and immunotherapy (**Table 1**). 16S rDNA sequencing or metagenomic shotgun sequencing (MSS) were used to analyze the composition of gut microbiota.

**Table 1:** Clinical evidence linking gut microbiota and cancer immunotherapy.

Cancer type	Therapy	Sample size	Alpha diversity	Bacteria related to response	Ref.
Metastatic melanoma	Anti-CTLA-4	26	Not mentioned	*Faecalibacterium prausnitzii*, *Gemmiger formicilis*, butyrate-producing bacteria SS2-1, *Ruminococcus*, Lachnospiraceae, *Clostridium* XIVa, *Blautia*	(71)
Metastatic melanoma	Anti-CTLA-4	38	Not mentioned	*Faecalibacterium*, *Gemminger*	(63)
Metastatic melanoma	ICI	39	No significant difference	*F. prausnitzii*, *Bacteroides thetaiotaomicron*, *Holdemania filiformis*, *Bacteroides caccae*	(72)
Metastatic melanoma	Anti-PD1	43	Higher in responders	*F. prausnitzii*, *Ruminococcus bromii*, *Porphyromonas pasteri*, *Clostridium hungati*, *Phascolarctobacterium faecium*	(73)
Metastatic melanoma	Anti-PD1 or anti-CTLA4	42	Not mentioned	*Enterococcus faecium*, *Collinsella aerofaciens*, *Bifidobacterium adolescentis*, *Klebsiella pneumoniae*, *Veillonella parvula*, *Parabacteroides merdae*, *Lactobacillus* sp., *Bifidobacterium longum*	(74)
Metastatic melanoma	ICI	25	No significant difference	*Streptococcus parasanguinis*, *Bacteroides massiliensis*	(75)
NSCLC	Anti-PD1	25	Higher in responders	*Alistipes putredinis*, *B. longum*, *Prevotella copri*	(76)
NSCLC	Anti-PD1/PD-L1	70	Higher in responders	Clostridiales, Ruminococcaceae *UCG 13*	(77)
NSCLC	ICI	17	No significant difference	*Lactobacillus*, *Clostridium*, *Syntrophococcus*	(78)
NSCLC	Anti-PD1	63	No significant difference	*Parabacteroides*, *Methanobrevibacter*	(79)
NSCLC and gastric cancer	Anti-PD1	38	Higher in responders	Ruminococcaceae	(80)
NSCLC and RCC	Anti-PD1	100	Not mentioned	*Akkermansia muciniphila*, Lachnospiraceae, *Erysipelotrichaceae bacterium*, *E. faecium*, *Alistipes indistinctus*, *B. caccae*, *Bacteroides xylanisolvens*, *Bacteroides nordii*	(81)
RCC	Anti-PD1	22	No significant difference	*Akkermansia*	(82)
Solid tumors	Chemotherapy/immunotherapy	26	Higher in responders	*B. xylanisolvens*, *Bacteroides ovatus*, *P. copri*, *Alistipes* spp.	(83)
Thoracic neoplasms	Anti-PD1	42	No significant difference	Akkermansiaceae, Enterococcaceae, Enterobacteriaceae, Carnobacteriaceae, Clostridiales Family XI	(84)
Gastric cancer	Anti-PD1	501	Higher in responders	*Odoribacter*, *Veillonella*	
Gastrointestinal cancer	Anti-PD1/PD-L1	74	No significant difference	*Prevotella*, Ruminococcaceae, Lachnospiraceae	(65)
Hepatocellular carcinoma	Anti-PD1	8	Higher in responders	*A. muciniphila*, Ruminococcaceae spp., *Bifidobacterium dentium*, *Lactobacillus*	(85)

NSCLC, non-small cell lung carcinoma; ICI, immune checkpoint inhibitor; RCC, renal cell carcinoma.

ICIs were first approved by the FDA to cure melanoma. The response of melanoma to ICIs was associated with a range of factors; integrative molecular and clinical modeling was used to predict the response (86). Since 2017, there have been six clinical studies, including 213 patients, that took insight into the association between the gut microbiota and the immunotherapy on metastasis melanoma (63, 71–75). All of them took the baseline (prior ICI treatment) microbiota into the first consideration. Totally, 26 bacteria were found by those studies to be related to a positive response in metastatic melanoma patients, including longer PFS and overall survival (OS). Among those bacteria, *Faecalibacterium prausnitzii* was found enriched in responders by three studies, from the United States and France, respectively (71–73). In addition, Coutzac et al. revealed that responders had increased *Faecalibacterium* (63). Three species of *Bacteroides* (72, 75), two species of *Bifidobacterium* (74), a species of *Clostridium* (71, 73) correlated with immunotherapy response positively. *Gemmiger formicilis* (71) and *Gemminger* (63), *Ruminococcus bromii* (73), and *Ruminococcus* (71) were reported to be enriched in responders. However, only Gopalakrishnan et al. (73) showed increased alpha diversity in responders.

The anti-PD-1/PD-L1 treatment has become the first-line strategy for NSCLC. There were four clinical studies (76–79) about gut microbiota and ICI focused on NSCLC patients, and another two (80, 81) included NSCLC patients. All of those studies recruited East Asian patients, except for Routy et al. (81). In addition, Katayama et al. (78) and Jin et al. (76) took the progression (during ICI treatment) microbiota into consideration. In general, a positive correlation between alpha diversity and ICI response was found in three of the six clinical studies (76, 77, 80). Nineteen bacteria were related with a positive response, including *Bacteroides* (81), *Bifidobacterium* (76), *Clostridium* (78), and *Ruminococcus* (78, 80), which correlated with immunotherapy response on metastasis melanoma positively, but *Faecalibacterium* was not in the NSCLC list.

In addition, there are some studies that revealed the association between gut microbiota and immunotherapy in the other solid tumor models (65, 80–85). Three of those clinical studies found the responders with higher alpha diversity (80, 83, 85). Worth mentioning is a clinical study involving 501 patients that also revealed the positive correlation between alpha diversity and ICI response (https://meetinglibrary.asco.org/record/193964/abstract). Similar to results of metastasis melanoma and NSCLC, *Bacteroides* (81, 83), *Bifidobacterium* (85), and *Ruminococcus* (65, 85) were enriched in the responders on other solid tumors. Furthermore, *Akkermansia muciniphila* was found enriched in responders by two individual studies (81, 85); Agarwal et al. (82) and Yin et al. (84) showed that *Akkermansia* correlated with beneficial response.

In summary, many clinical studies identified the association between gut microbiota and immunotherapy. Although various sample volumes from different regions, different collection techniques, cancer types, and distinctive sequencing methods limit the accuracy of gut microbial comparisons, we can find some clues from those studies. First, although only a part of the studies showed a positive correlation between alpha diversity and immunotherapy response, none of them showed a negative correlation, indicating the importance of alpha diversity. Second, *Bacteroides*, *Bifidobacterium*, *Ruminococcus*, and *Akkermansia* were frequently found to be associated with beneficial responses, indicating that they may play a role in regulating immunotherapy. Regrettably, there are no available data showing the association between gut microbiota and the efficiency of neoadjuvant immunotherapy. However, Batten et al. revealed that the diversity and composition of gut microbiota were associated with immune-related adverse events in neoadjuvant immunotherapy (87). Rajji et al. reported that antibiotics were associated with less benefit from neoadjuvant immunotherapy on bladder cancer (88).

#### 2.1.3 Mouse Models Showing the Effect of Gut Microbiota on Immunotherapy

There is no available clinical trial that shows the effect of gut microbiota on immunotherapy; therefore, the effect was illustrated by mouse models only (**Table 2**).

**Table 2:** Gut microbiota enhancing cancer immunotherapy in mice.

Tumor model	Therapy	Beneficial bacteria species	Specific mechanisms	Ref.
B16 melanoma	Anti-PD-L1	*Bifidobacterium*	DC, IFNγ^+^CD8^+^ T cells	(41)
B16 SIY melanoma	Anti-PD-L1	Responder patient FMT	CD8^+^ T cells	(74)
RET melanoma	Anti-PD-1	*Akkermansia muciniphila*, *Alistipes*, *Enterococcus hirae*	CCR9^+^CXCR3^+^CD4^+^ T cells *via* IL-12	(81)
MC38 colon	Anti-CD47	*Bifidobacterium*	DC *via* STING	(28)
MC38 colon	Anti-IL-10 + CpG	*Alistipes shahii*, *Ruminococcus*	TNF^+^ myeloid cell	(43)
MC38 colon	Anti-CTLA4	*Bifidobacterium pseudolongum*, *Lactobacillus johnsonii*, *Olsenella* species	Th1 cell, IFNγ^+^CD8^+^ T cells	(34)
MC38 colon	Anti-PD-1 or Anti-CTLA4	*Ruthenibacterium lactatiformans*, *Eubacterium limosum*, *Fusobacterium ulcerans*, *Phascolarctobacterium succinatutens*, *Bacteroides uniformis*, *Bacteroides dorei*, *Paraprevotella xylaniphila*, *Parabacteroides distasonis*, *Parabacteroides johnsonii*, *Parabacteroides gordonii*, and *Alistipes senegalensis*	CD103^+^ DC, IFNγ^+^CD8^+^ T cell	(89)
MC38 colon	Anti-PD-1	*Bacteroides fragilis*, a non-enterotoxigenic species, *Erysipelatoclostridium ramosum*, and *Alistipes onderdonkii*	CD103^+^CD11b^−^ DC	(90)
MCA205 sarcoma	Anti-CTLA4	*B. fragilis*, *Bacteroides thetaiotaomicron*, *Burkholderia*	Memory T cell	(51)
MCA205 sarcoma	Anti-PD-1	Responder patient FMT, *A. muciniphila*, *E. hirae*, *Alistipes*	CCR9^+^CXCR3^+^CD4^+^ T cells *via* IL-12	(81)
RENCA RCC	Anti-PD-1 + anti-CTLA4	Responder patient FMT	CCR9^+^CXCR3^+^CD4^+^ T cells *via* IL-12	(81)
LLC lung carcinoma	Anti-PD-1	*A. muciniphila*, *Alistipes*, *E. hirae*	CCR9^+^CXCR3^+^CD4^+^ T cells *via* IL-12	(81)

RCC, renal cell carcinoma; DC, dendritic cell; FMT, fecal microbiota transplant; STING, stimulator of interferon genes; LLC, Lewis lung carcinoma.

First of all, mice with different gut microbiota show distinct responses to immunotherapy. Wild-type (WT) mice from Jackson Laboratory (JAX) and Taconic Biosciences (TAC) were reported to have a distinct gut microbiome that contributes to their distinct immune signatures (91). The JAX mice carrying B16 melanoma and MC38 showed enhanced response to anti-PD-L1 and anti-CD47, respectively, compared with TAC mice (28, 41). Besides, loss of gut microbiota by using GF mice or treating specific pathogen-free (SPF) mice with antibiotics ablated the response to immunotherapies, including anti-IL-10 receptor plus CpG-oligonucleotide on MC38 tumor-bearing mice (43), anti-CTLA4 on MCA205 sarcoma-bearing mice (51), anti-CTLA4 or anti-PD1 on MC38 tumor-bearing mice (34, 89), anti-CD47 on MC38 tumor-bearing mice (28), and anti-PD1 on CT26 tumor-bearing mice (92). This phenomenon was a window that revealed the association of gut microbiota with immunotherapy.

Second, when given different gut microbiota, gnotobiotic mice appeared to have a distinct response to immunotherapy, which demonstrated the effect of gut microbiota on immunotherapy, as well. As mentioned above, Matson et al. (74) investigated the gut microbiota of 38 metastatic melanoma patients treated with anti-PD1 and found the difference. Reconstitution of GF mice with fecal material from responding patients could lead to improved tumor control, augmented T-cell responses, and greater efficacy of anti-PD-L1 therapy on the B16 melanoma mouse model (74). In addition, a study by Routy et al. exhibited the same benefit from responders’ gut microbiota on MCA205 sarcoma (81).

Last but not least, the beneficial effects of defined bacteria on immunotherapy have also been demonstrated by mouse models. Oral supplementation with *Alistipes shahii* or *Ruminococcus* reversed immunotherapy inhibition by the antibiotic treatment, but not *Lactobacillus fermentum* (43). Gavage of TAC mice with *Bifidobacterium* species enhanced the effect of anti-PD-L1 on MC38 colon cancer, with DC activation and increased IFNγ producing CD8^+^ T cells (41). As well, *Bifidobacterium* species enhanced the effect of anti-CD47 (28). CD47, known as the “don’t eat me” signal, is the phagocytosis checkpoint as a new target for cancer immunotherapy (93). *Bacteroides fragilis*, *Bacteroides thetaiotaomicron*, and *Burkholderia* effectively aided immunotherapy of anti-CTLA4 on MCA205 sarcomas depending on intratumoral CD11b^+^ DCs secreting IL-12 and splenic ICOS^+^ Ki67^+^ IFNγ^+^ TNFα^+^ T cells, and tumor-infiltrating T cells, but not *Parabacteroides distasonis* nor *Escherichia coli* nor *Bacteroides uniformis* (51). Oral gavage with *A. muciniphila* after FMT with non-responder feces restored the efficacy of anti-PD-1 on orthotopic Lewis lung carcinoma (LLC) non-small cell lung cancers and MCA205 mouse models, which depended on IL-12, with increasing recruitment of CCR9^+^CXCR3^+^CD4^+^ T cell into mouse tumor, in the mechanism (81). A similar phenotype was also revealed on renal cell carcinoma (RCC) tumor-bearing mice (94). Tanoue et al. (89) isolated 11 human gut bacteria that increased colonic IFNγ^+^ T cells, including *Ruthenibacterium lactatiformans*, *Eubacterium limosum*, *Fusobacterium ulcerans*, *Phascolarctobacterium succinatutens*, *B. uniformis*, *Bacteroides dorei*, *Paraprevotella xylaniphila*, *P. distasonis*, *Parabacteroides johnsonii*, *Parabacteroides gordonii*, and *Alistipes senegalensis*. Administration with the 11-bacterium mix (11-mix) recovered efficacy of anti-PD1 or anti-CTLA4 with infiltration with IFNγ^+^ T cells in MC38 tumor. Mager et al. (34) showed that *Bifidobacterium pseudolongum*, *Lactobacillus johnsonii*, and *Olsenella* species significantly enhanced efficacy of anti-CTLA4 on MC38 model and azoxymethane/dextran sodium sulfate (AOM/DSS) model, with increased IFNγ^+^CD8^+^ T cells and IFNγ^+^ CD4^+^ T cells. Roberti et al. (90) found four immunogenic bacteria (*B. fragilis*, a non-enterotoxigenic species, *Erysipelatoclostridium ramosum*, and *Alistipes onderdonkii*), which were able to boost vaccine (oxaliplatin-exposed organoids) efficacy and anti-PD1 efficacy on MC38, in a CD103^+^CD11b^−^DC [conventional type 1 DCs (cDC1)]-dependent manner.

In summary, *via* mouse models, the causal/mechanistic link between gut microbiota and immunotherapy was illustrated. *Bacteroides*, *Bifidobacterium*, *Ruminococcus*, *Lactobacillus*, *Enterococcus*, and *Akkermansia*, associated with the response to immunotherapy in clinical studies, were revealed to activate immunity and boost the efficiency of immunotherapy in mouse models.

### 2.2 Gut Microbiota and Immune Response in Chemotherapy

Although not traditionally considered as immunotherapy, effective chemotherapy is also dependent on intact immune responses; therefore, the effect of gut microbiota on conventional chemotherapy depends on modulating the immune response.

Cyclophosphamide, a prominent alkylating anticancer agent, inhibits tumor outgrowth by inducing immunogenic cancer cell death (95, 96), reverting immunosuppressive T cells (97), and promoting Th1 and Th17 cells (98). GF or antibiotic-treated mice carrying MCA205 sarcoma lost cyclophosphamide tumor inhibition, suggesting that the gut microbiota plays a critical role in controlling cancer during cyclophosphamide treatment. While oral gavage with *E. hirae* clone 13144 and *Barnesiella intestinihominis* reinstated cyclophosphamide efficacy, but not *P. distasonis*, *Lactobacillus plantarum*, *Lactobacillus reuteri*, and *L. johnsonii*, which segmented filamentous bacteria, even other *E. hirae* isolates (37, 38, 53). In mechanism, *E. hirae* clone 13144 translocated into secondary lymphoid organs, where they stimulated the generation of a specific subset of “pathogenic” Th17 cells and memory Th1 immune responses, which cross-react with tumor-associated antigens. Finally, *E. hirae* clone 13144 increased the intratumoral CD8^+^/Treg ratio and enhanced chemotherapy (37, 38, 53). *Ba. intestinihominis* raised chemotherapy of cyclophosphamide through yielding tumor IFNγ T-cell infiltration (37).

## 3 Strategies to Improve Gut Microbiota in Cancer Immunotherapy

### 3.1 Fecal Microbiota Transplant

FMT is when stool from a healthy donor is made into a liquid mixture and transferred into the gut of a different person to try to reintroduce or boost helpful organisms, which represents the most direct means to manipulate the gut microbiota. Based on results from preclinical studies discussed above, FMT is considered as an intervention to treat patients undergoing immunotherapy, especially those administered with ICIs, aiming for the safety and response of the combo of FMT and immunotherapy. Currently, melanoma, prostate cancer, gastrointestinal system cancer, NSCLC, and mesothelioma are enrolled by several FMT-related clinical trials (**Table 3**).

**Table 3:** Clinical trials linking gut microbiota and cancer immunotherapy.

Interventions	Trial number	Conditions	Major microbiota and immune related outcomes	Phases
**FMT**				
FMT *via* colonoscopy	NCT04264975	Solid carcinoma	Response to immunotherapy plus FMT	Not applicable
FMT *via* colonoscopy	NCT04056026	Mesothelioma	Response to Keytruda plus FMT	Early Phase 1
FMT *via* colonoscopy	NCT03772899	Melanoma	Response to immunotherapy plus FMT	Phase 1
FMT *via* endoscopy	NCT04116775	Metastatic castration-resistant prostate cancer	Response to pembrolizumab plus FMT	Phase 2
FMT *via* oral capsule	NCT04130763	Gastrointestinal system cancer	Response to anti-PD-1 plus FMT	Phase 1
FMT *via* oral capsule	NCT04521075	Metastatic melanoma or NSCLC	Response to nivolumab plus FMT	Phase 1/Phase 2
FMT *via* colonoscopy and oral capsule	NCT03353402	Melanoma Stage IV and unresectable Stage III	Response to immunotherapy plus FMT	Phase 1
FMT	NCT04577729	Melanoma Stage III and IV	Response to checkpoint inhibitor plus FMT	Not applicable
FMT *via* colonoscopy	NCT03341143	PD-1 resistant/refractory melanoma	Response to checkpoint inhibitor plus FMT	Phase 2
**Diet**				
Fasting mimicking diet	NCT03454282	Breast cancer or melanoma	Tumor-infiltrating lymphocytes, gut microbiota composition	Not applicable
Dietary supplement: IGEN0206	NCT04009122	Non-small cell lung cancer metastatic	Quality of life, changes in the microbiota, interleukin levels, cytokines levels	Not applicable
**Probiotics**				
Oral Primal Defense Ultra Probiotic Formula	NCT03358511	Breast cancer	Mean number of cytotoxic T cell	Not applicable
Oral MRx0518	NCT04193904	Pancreatic cancer	Tumor infiltrating lymphocytes	Phase 1
5	NCT03817125	Metastatic melanoma	Response to checkpoint inhibitor	Phase 1
Oral MET-4	NCT03838601	Head and neck squamous cell carcinoma	Bacterial composition and diversity, blood immune cell profiling	not applicable
Oral BB536, LA1	NCT00936572	Colorectal cancer	Immune and inflammatory response, bacterial translocation	Phase 2
IV JNJ-64041809	NCT02625857	Metastatic castration-resistant prostate cancer	Immune responses	Phase 1
Oral MRx0518	NCT03637803	Solid tumors	Clinical benefit of MRx0518 in combination with pembrolizumab	Phase 1/Phase 2
Oral RBX7455	NCT04139993	Breast cancer	Intratumoral immunomodulatory	Early Phase 1
Oral GEN-001	NCT04601402	Solid tumors	Response to avelumab	Phase 1
Oral EDP1503	NCT03595683	Melanoma	Response to pembrolizumab	Phase 2
Oral MET-4	NCT03686202	Solid tumors	Relative abundance of immunotherapy-responsiveness associated species of MET-4	Early Phase 1
Oral VE800	NCT04208958	Selected types of advanced or metastatic cancer	Safety and efficacy of VE800 in combination with nivolumab	Phase 1/Phase 2
Oral MRx0518	NCT03934827	Solid tumors	Safety, tolerability, and immune system modulation of MRx0518	Phase 1
Oral EDP1503	NCT03775850	Colorectal cancer, breast cancer, and checkpoint inhibitor relapsed tumors	Safety, tolerability, and efficacy of EDP1503 alone and in combination with pembrolizumab	Phase 1/Phase 2
**Antibiotic**				
Oral vancomycin	NCT03785210	Refractory primary hepatocellular carcinoma or liver-dominant metastatic cancer from colorectal or pancreatic cancers	Response to nivolumab	Phase 2

FMT, fecal microbiota transplant; NSCLC, non-small cell lung carcinoma.

The key factor of those clinical trials is the criteria of the donor. Six of nine clinical trials treated patients who respond to immunotherapy as donors (NCT04264975, NCT04116775, NCT04521075, NCT03353402, NCT04577729, and NCT03341143). Recently, the result of the phase 1 clinical trial (NCT03353402) was published (99). To assess the safety and feasibility of fecal FMT and re-induction of anti-PD-1 immunotherapy, the trial recruited 10 patients with anti-PD-1-refractory metastatic melanoma. Two FMT donors were included in the trials who had previously been treated with anti-PD-1 monotherapy and achieved a complete response. First of all, the gut microbiota of all recipients significantly differed from their baseline and closed to the donors. In detail, patients who received donor #1 sample had a greater relative abundance of *Ruminococcus* and *Bifidobacterium adolescentis*, whereas those who received donor #2 sample had an overrepresentation of Clostridiaceae (99). In addition, treatment increased multiple immune-related gene sets in the tumor tissue of donor #1 group, including IFNγ-mediated signaling pathway, T-cell activation, MHC-II protein complex, DC differentiation, and Th1-type immune response (99). Most importantly, three of 10 recipients achieved objective responses, all of them from donor #1 group, and only one recipient had a mild temporary bloating considered as an FMT-related adverse event (99). Another phase 2 clinical trial (NCT03341143) showed that six of 15 PD-1-refractory patients with melanoma benefited from the FMT (100). In this study, seven donors were included, including four with a complete response and three with a partial response. Responders’ recipient microbiota exhibited a significant shift toward the donor composition compared with the non-responders’. Successful FMT was enriched in Ruminococcaceae, Bifidobacteriaceae, and Lachnospiraceae. A coinciding immune activity after the FMT was found in blood and tumor microenvironment (100). Three of nine clinical trials treated healthy people as donors (NCT04056026, NCT03772899, and NCT04130763). Interestingly, the activation of immune response was also found in advanced or metastatic melanoma patients with FMT from healthy donors (NCT03772899) (101). Most importantly, these three published trials showed a favorable safety profile and represented the first clinical evidence that the gut microbiota may have an impact on antitumor immunity and potentially even responses to immunotherapies.

Besides the criteria of donors, those clinical trials differed on the FMT preparations (**Table 3**). Generally, FMT preparations can be performed *via* oral administration of lyophilized or frozen pills and capsules, or direct delivery by endoscopy. The lower routes of administration (colonoscopy or enema) appeared to be more successful than the upper routes (gastroscopy, or nasogastric and nasointestinal tubes) (102). Maybe this is the reason that most of those clinical trials administer FMT with colonoscopy.

However, to translate FMT into the clinic, there are a number of problems that we need to face. First of all is the safety issue. FDA has reported safety alerts after the death of patients receiving FMT for *Clostridium difficile* infection who developed infections caused by enteropathogenic bacteria contained in the FMT. Besides harmful bacteria, the harmful virus should also be screened before FMT, considering the intestinal epithelium is a tropism of SARS-CoV-2. Second issue is how to define the optimal donors. Several investigators recruit donors from patients who previously responded to immunotherapy, while others prefer healthy volunteers. Now only three positive results have been published. Two of them showed the benefits from responding patients, and one of them showed the benefits from healthy people. Considering that most studies revealed the difference of gut microbiota between responders and non-responders, it seems that responding patients should be better donors. In addition, the kinds of pathologies of the donor should be excluded. In one case, the obese phenotype has been transferred from a donor to a recipient (103). Last, FMT may benefit from host conditioning, including diet, probiotics, and antibiotics. Further studies are needed to make a synergetic combo of the FMT and host conditioning.

### 3.2 Diet

As a dominant determinant of interindividual microbiota variation (104, 105), diet is the key determinant of the microbiota configuration, through modulation of the abundance of microbial species and their individual or collective functions (106–108). Hippocrates noted “Let food be thy medicine and medicine be thy food.” Owing to the advantageous safety, cost, and availability, diet could be a promising clinical intervention to modulate gut microbiota and downstream immune in cancer patient populations.

Prebiotics are a source of diet for your gut’s healthy bacteria. They are carbs that our body cannot digest. The well-known prebiotics, microbiota-accessible carbohydrates, have a major impact on gut microbiota composition, diversity, and richness (109). Microbiota-accessible carbohydrates are fermented by gut microbiota to produce SCFAs, which have been discussed above in modulating immunotherapy. It benefited the exclusion of pathogens such as *Citrobacter rodentium* and *C. difficile* (110, 111). Another prebiotic, plant polysaccharide inulin, increased both *Faecalibacterium* and *Bifidobacterium* species in gut microbiota, which are considered potentially favorable for immunotherapy (112). The effect of a dietary supplemental nutritional product (IGEN0206) on the quality of life, nutritional status, and shift in the gut microbiota of patients with NSCLC was investigated by a clinical study (NCT04552418). Unfortunately, we have no idea of the prebiotics in IGEN0206.

Besides prebiotics, the main components of diets shift gut microbiota and immunity, including calorie, protein, and fat. A plant-based, calorie-restricted, low-protein diet, also known as fasting mimicking diet (FMD), modulated gut microbiota composition and immune cell profiles to reduce inflammatory bowel disease pathology (113). It has been proposed as a potential anticancer dietary intervention by enhancing cytotoxic CD8^+^ tumor-infiltrating lymphocytes (114). At present, NCT03454282 is designed to explore the impact of FMD on the gut microbiota composition, peripheral blood mononuclear cells, tumor-infiltrating lymphocytes, and metabolic parameters of breast cancer or melanoma patients.

The population structure responds to acute dietary change, as evidenced by rapid and substantial increases in populations at the genus and species levels. However, dietary change does not necessarily induce a permanent compositional shift, at least at the phylum level, although evidence for this assertion is limited (115). As a result, diets might not able to reshape the gut microbiota as dramatically as FMT. But the advantage in safety and convenience of diets is obvious. Considering the restricted effect of diets on gut microbiota, the combination of diets and FMT might give their advantages a full play to modulate gut microbiota and immunotherapy.

### 3.3 Probiotics

Beneficial or immune-modulating bacteria could be administered as a probiotic to manipulate cancer immunotherapy. Probiotics could provide a more feasible method of microbial manipulation in the clinical setting. Many clinical trials using probiotics in cancer patients have been initiated with some completed (**Table 3**).

Most of the probiotics are composed of single strains. MRx0518 is a strain of *Enterococcus gallinarum*, isolated from a healthy human fecal sample (116). EDP1503 is a strain of *Bifidobacterium animalis* subsp. *lactis*, BB536 is a strain of *Bifidobacterium longum*, and LA1 is a strain of *Lactobacillus acidophilus*. JNJ-64041809 is a live attenuated, double-deleted *Listeria* administered intravenously. GEN-001 is a single-strain bacteria isolated from the gut of healthy human volunteers. As mentioned above, *Enterococcus*, *Bifidobacterium*, and *Lactobacillus* are related to immunotherapy (74, 76, 81, 85). Especially, *Bifidobacterium* species have been demonstrated to enhance the response to ICIs in animal models by several studies (28, 34, 41). Currently, initial data from the first six patients of NCT03637803 showed that MRx0518 combined with pembrolizumab is well tolerated in patients with solid tumors who have developed resistance to anti-PD-1/PD-L1. Two patients have shown a partial response with evidence of increased tumor-infiltrating lymphocytes, according to the RECIST v1.1 criteria 1. One additional patient has a stable disease. No drug-related serious adverse events have been noted (https://www.londonstockexchange.com/news-article/DDDD/clinical-observations-from-mrx0518/14295955). Initial data of NCT03775850 show that an overall response rate (ORR) of 25% (2/8) and a disease control rate of 37.5% (3/8) were observed across all triple negative breast cancer (TNBC) subjects receiving high-dose EDP1503. ORR was 33% (2/6) among response-evaluable patients on the high dose, with two patients awaiting first response assessment. Historic studies of anti-PD-1 monotherapy in heavily pretreated TNBC patients have yielded an ORR of 5%–10% (https://ir.evelobio.com/news-releases/news-release-details/evelo-biosciences-present-clinical-data-phase-12-trial-edp1503). NCT02625857 showed that JNJ-64041809 has a manageable safety profile and activation of the immune response. Nevertheless, observed immune activation with monotherapy did not translate into clinical activity (117).

The probiotics could also be a consortium of live bacteria, including Primal Defense Ultra Probiotic Formula, SER-401, and MET-4 and VE800. VE800, which consisted of 11 clonal human commensal bacteria strains, activated the immunotherapy *via* CD8^+^ T cells in animal models (89). The consortium seems more powerful in shifting gut microbiota than single bacteria; however, it is a pity that there are no available clinical data to show the safety of probiotic consortiums, although five clinical trials are going to reveal the safety and clinical response.

In addition to the strain isolated from humans, synthetically engineered microorganisms can also be implanted as probiotics. Advances in synthetic biology are enabling the design of microorganisms based on therapeutic needs. Currently, engineered a non-pathogenic *E. coli* strain, expressing encoded nanobody antagonist of CD47, or nanobodies targeting PD-L1 and CTLA4, or STING agonist, were administered to activate systemic antitumor immunity and to regress tumor burden in mouse models (118–120), although, until now, those engineered probiotics were designed to kill tumors, directly. Along with a deep understanding of the role of gut microbiota in cancer immunotherapy, engineered probiotics will be applied to modulating gut microbiota, as an adjuvant of immunotherapy.

We believe that probiotics are the future to improve gut microbiota for immunotherapy. Compared with FMT, probiotics do not need donors nor the criteria for donors. In addition, probiotics contain less harmful and dispensable matter. Last, probiotics are easier for the industry. However, a deeper understanding of the mechanism between gut microbiota and immunotherapy is needed to develop immunotherapeutic probiotics.

### 3.4 Antibiotics

Antibiotic administration is another straightforward intervention to module gut microbiota and the downstream cancer immunotherapies. By removing harmful bacteria, some antibiotics can provide a positive effect on the gut microbiota and immunotherapy. Vancomycin targets gram-positive bacteria, including butyrate-producing bacteria and decreasing SCFA concentrations. Vancomycin treatment induced an increase of systemic CD8α^+^ DCs, tumor-associated antigen cross-priming with antitumor CD8^+^ T cell elicitation, and tumor growth inhibition in mice, *via* decreasing SCFAs (121, 122). Recently, a phase 2 single-arm clinical trial (NCT03785210) was designed to investigate if nivolumab given with tadalafil and vancomycin causes liver tumor to shrink (**Table 3**).

Nevertheless, antibiotic classes should be carefully considered. Due to the lack of specificity, antibiotics decrease bacterial diversity, eliminate beneficial bacteria, and give rise to dysbiosis. As a matter of fact, numerous clinical studies from France, China, Japan, Canada, and the United States unleashed antibiotic treatment prior to immunotherapy was associated with reduced clinical benefit on melanoma (123), NSCLC (81, 124–127), and RCC (81, 94, 124, 128). All of those studies found that patients with antibiotic treatment prior to immunotherapy had decreased diversity of gut microbiota and worse PFS and OS. To some extent, the results are consistent with the investigation of responders and non-responders showing a positive correlation between alpha diversity and immunotherapy response. On the other hand, taking the advantage of broad-spectrum depletion of naïve gut microbiota, antibiotics could be used before FMT to achieve better microbial modulation. For instance, Baruch et al. (99) treated patients with vancomycin and neomycin to deplete their own native microbiota before FMT *via* colonoscopy and *via* oral capsules.

## 4 Discussion

The dynamic nature of the microbiota makes it an attractive target for therapeutic intervention in a range of conditions, as engraftment or elimination of particular microorganisms. The shift of gut microbiota contributes to altering both innate and adaptive immunity. In addition, many studies incorporating preclinical and clinical studies have gained our insight into the influence of gut microbiota on cancer immunotherapy. Via MAMPs, microbial metabolites, and molecular mimicry, the gut microbiota educates both local and systematic immunity to alter the response to cancer immunotherapy. Therefore, in the age of microbiome, therapeutic strategies targeting gut microbiota, including FMT, diet, probiotics, and antibiotics, are developed to enhance responses to cancer immunotherapy. However, there is still a great deal to investigate the inherent mechanisms, as well as optimal strategies.

To identify causal host–microbiota relationships and mechanisms, there are two approaches generally, the microbiota-based approach and the molecule-based approach (129). The microbiota-based approach is the more often used. First, a complex microbiota is found to promote a given phenotype. Then several methods, including 16S DNA sequence, antibiotic treatment, and *in vitro* culture, are used to narrow down the entire microbiota to a single effector species or consortium. Furthermore, single species intervention and/or bacterial genetic engineering studies are performed to uncover the mechanisms. The molecule-based approach starts from a small molecule, which is proven to promote a given phenotype. Then by searching genomic databases or the literature, the biosynthetic machinery of the molecules and the functional species will be identified. Because many metabolic pathways are conserved in bacteria, the molecule-based approach bacteria may be possible to identify several effector species. If necessary, further investigations are needed to identify the most critical species. Up to now, nearly all of the mechanism studies focusing on the role of gut microbiota in response to immunotherapy belong to microbiota-based approaches (28, 34, 37, 41, 43). No study used a molecule-based approach to explore the host–microbiota relationships in cancer immunotherapy. Given the fact that there are many sensitive *in vitro* models in investigating cancer immunotherapy (130, 131), systematic screening of microbiota-derived molecules with those models is an effective method to identify the molecules associated with the given phenotype. Furthermore, the systematic screen will provide one or more great starts for the molecule-based approach in further revealing the inherent mechanisms. Therefore, the molecule-based approach should be a window to explore causal host–microbiota relationships and mechanisms in cancer immunotherapy.

**Figure 2:**
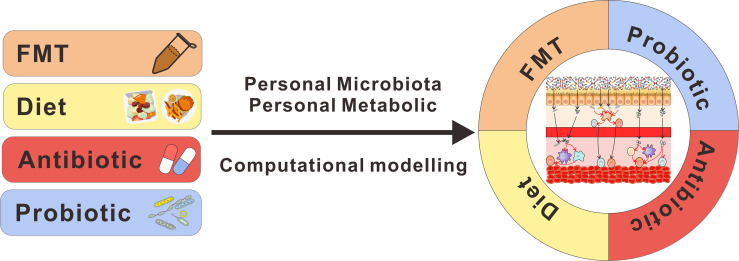
Design of microbial therapeutics to benefit cancer immunotherapy. Stable microbial engraftment is manipulated by intrinsic microbiota, extrinsic nutrients, and colonic metabolic. As a tool, computational modeling can be used to predict the engraftment of microbiota members. Thus, with the deeper insight into this field, the rational design of microbial therapeutics will take all of those factors into consideration and combine those individual therapeutic strategies to improve cancer immunotherapy.

Furthermore, additional complexities exist as we move forward with optimal microbiota-based strategies to improve therapeutic responses. First, although these clinical studies drew similar conclusions those clinical studies linking gut microbiota and immunotherapy and some beneficial bacteria have been identified by clinical and preclinical studies, there were diverse results; it is not very clear what composition of the gut microbiome is optimal to facilitate antitumor immunity. More researches should be performed to define the ideal gut microbiota for immunotherapy. Second, although there are various range of therapeutic options to shift gut microbiota, precise modulation with gut microbiota remains difficult owing to the interindividual heterogeneity inherent in humans. Computational models could help in the precise design of microbial therapeutics, which can be used to predict the engraftment of immunomodulatory microbiota members (132). Based on taxonomic analysis of gut microbiota, machine learning can provide new insights to predict disease states and outcomes, which is beneficial for personalized medicine (133). Last, stable microbial engraftment can be manipulated by intrinsic microbiota, extrinsic nutrients (134, 135), colonic metabolic state (136), and immune state (137). Thus, precision medicine interventions in gut microbiota and a rational combo of those individual therapeutic strategies are required to optimize to match the genetic, microbial, and metabolic profiles (**Figure 2**). Unfortunately, we lack the ability to reliably predict how these factors influence bacteria and their immunomodulatory properties, currently. Although the promise of microbial therapy has been revealed in cancer immunotherapy, a number of further studies are still needed to optimize therapeutic strategies.

## Author Contributions

BL, TG, and YH wrote the paper. XZ and LC revised the paper. All authors contributed to the article and approved the submitted version.

## Funding

This study was supported by the National Natural Science Foundation of China 81870759 (LC) and 82071106 (LC).

## Conflict of Interest

The authors declare that the research was conducted in the absence of any commercial or financial relationships that could be construed as a potential conflict of interest.

## Publisher’s Note

All claims expressed in this article are solely those of the authors and do not necessarily represent those of their affiliated organizations, or those of the publisher, the editors and the reviewers. Any product that may be evaluated in this article, or claim that may be made by its manufacturer, is not guaranteed or endorsed by the publisher.

## References

[1] Duffy MJ Crown J. Biomarkers for Predicting Response to Immunotherapy With Immune Checkpoint Inhibitors in Cancer Patients. Clin Chem (2019) 65:1228–38. doi: 10.1373/clinchem.2019.303644 31315901

[2] Ost KS Round JL. Communication Between the Microbiota and Mammalian Immunity. Annu Rev Microbiol (2018) 72:399–422. doi: 10.1146/annurev-micro-090817-062307 29927706 PMC7294967

[3] Thaiss CA Zmora N Levy M Elinav E. The Microbiome and Innate Immunity. Nature (2016) 535:65–74. doi: 10.1038/nature18847 27383981

[4] Fessler J Matson V Gajewski TF. Exploring the Emerging Role of the Microbiome in Cancer Immunotherapy. J Immunother Cancer (2019) 7:108. doi: 10.1186/s40425-019-0574-4 30995949 PMC6471869

[5] Schreiber RD Old LJ Smyth MJ. Cancer Immunoediting: Integrating Immunity’s Roles in Cancer Suppression and Promotion. Sci (New York NY) (2011) 331:1565–70. doi: 10.1126/science.1203486 21436444

[6] Sharma P Allison JP. The Future of Immune Checkpoint Therapy. Sci (New York NY) (2015) 348:56–61. doi: 10.1126/science.aaa8172 25838373

[7] Zappasodi R Merghoub T Wolchok JD. Emerging Concepts for Immune Checkpoint Blockade-Based Combination Therapies. Cancer Cell (2018) 33:581–98. doi: 10.1016/j.ccell.2018.03.005 PMC5896787 29634946

[8] Blank CU Rozeman EA Fanchi LF Sikorska K van de Wiel B Kvistborg P. Neoadjuvant *Versus* Adjuvant Ipilimumab Plus Nivolumab in Macroscopic Stage III Melanoma. Nat Med (2018) 24:1655–61. doi: 10.1038/s41591-018-0198-0 30297911

[9] Huang AC Orlowski RJ Xu X Mick R George SM Yan PK. A Single Dose of Neoadjuvant PD-1 Blockade Predicts Clinical Outcomes in Resectable Melanoma. Nat Med (2019) 25:454–61. doi: 10.1038/s41591-019-0357-y PMC6699626 30804515

[10] Rozeman EA Menzies AM van Akkooi ACJ Adhikari C Bierman C van de Wiel BA. Identification of the Optimal Combination Dosing Schedule of Neoadjuvant Ipilimumab Plus Nivolumab in Macroscopic Stage III Melanoma (OpACIN-Neo): A Multicentre, Phase 2, Randomised, Controlled Trial. Lancet Oncol (2019) 20:948–60. doi: 10.1016/S1470-2045(19)30151-2 31160251

[11] Ulas EB Dickhoff C Schneiders FL Senan S Bahce I. Neoadjuvant Immune Checkpoint Inhibitors in Resectable Non-Small-Cell Lung Cancer: A Systematic Review. ESMO Open (2021) 6:100244. doi: 10.1016/j.esmoop.2021.100244 34479033 PMC8414043

[12] Sahin U Oehm P Derhovanessian E Jabulowsky RA Vormehr M Gold M. An RNA Vaccine Drives Immunity in Checkpoint-Inhibitor-Treated Melanoma. Nature (2020) 585:107–12. doi: 10.1038/s41586-020-2537-9 32728218

[13] Champiat S Ferrara R Massard C Besse B Marabelle A Soria JC. Hyperprogressive Disease: Recognizing a Novel Pattern to Improve Patient Management. Nat Rev Clin Oncol (2018) 15:748–62. doi: 10.1038/s41571-018-0111-2 30361681

[14] Chen DS Mellman I. Elements of Cancer Immunity and the Cancer-Immune Set Point. Nature (2017) 541:321–30. doi: 10.1038/nature21349 28102259

[15] Tran L Theodorescu D. Determinants of Resistance to Checkpoint Inhibitors. Int J Mol Sci (2020) 21:1594. doi: 10.3390/ijms21051594 PMC7084564 32111080

[16] Kishton RJ Sukumar M Restifo NP. Metabolic Regulation of T Cell Longevity and Function in Tumor Immunotherapy. Cell Metab (2017) 26:94–109. doi: 10.1016/j.cmet.2017.06.016 28683298 PMC5543711

[17] Gopalakrishnan V Helmink BA Spencer CN Reuben A Wargo JA. The Influence of the Gut Microbiome on Cancer, Immunity, and Cancer Immunotherapy. Cancer Cell (2018) 33:570–80. doi: 10.1016/j.ccell.2018.03.015 PMC6529202 29634945

[18] Frankel AE Deshmukh S Reddy A Lightcap J Hayes M McClellan S. Cancer Immune Checkpoint Inhibitor Therapy and the Gut Microbiota. Integr Cancer Ther (2019) 18:1534735419846379. doi: 10.1177/1534735419846379 31014119 PMC6482659

[19] Thaiss CA Levy M Suez J Elinav E. The Interplay Between the Innate Immune System and the Microbiota. Curr Opin Immunol (2014) 26::41–48. doi: 10.1016/j.coi.2013.10.016 24556399

[20] Esser D Lange J Marinos G Sieber M Best L Prasse D. Functions of the Microbiota for the Physiology of Animal Metaorganisms. J Innate Immun (2019) 11:393–404. doi: 10.1159/000495115 30566939 PMC6738199

[21] Ley RE Peterson DA Gordon JI. Ecological and Evolutionary Forces Shaping Microbial Diversity in the Human Intestine. Cell (2006) 124:837–48. doi: 10.1016/j.cell.2006.02.017 16497592

[22] Qin J Li R Raes J Arumugam M Burgdorf KS Manichanh C. A Human Gut Microbial Gene Catalogue Established by Metagenomic Sequencing. Nature (2010) 464:59–65. doi: 10.1038/nature08821 20203603 PMC3779803

[23] Levy M Thaiss CA Elinav E. Metagenomic Cross-Talk: The Regulatory Interplay Between Immunogenomics and the Microbiome. Genome Med (2015) 7:120. doi: 10.1186/s13073-015-0249-9 26589591 PMC4654884

[24] Thaiss CA Levy M Itav S Elinav E. Integration of Innate Immune Signaling. Trends Immunol (2016) 37:84–101. doi: 10.1016/j.it.2015.12.003 26755064

[25] Hormann N Brandao I Jackel S Ens N Lillich M Walter U. Gut Microbial Colonization Orchestrates TLR2 Expression, Signaling and Epithelial Proliferation in the Small Intestinal Mucosa. PloS One (2014) 9:e113080. doi: 10.1371/journal.pone.0113080 25396415 PMC4232598

[26] Zhang D Chen G Manwani D Mortha A Xu C Faith JJ. Neutrophil Ageing is Regulated by the Microbiome. Nature (2015) 525:528–32. doi: 10.1038/nature15367 PMC4712631 26374999

[27] Clarke TB Davis KM Lysenko ES Zhou AY Yu Y Weiser JN. Recognition of Peptidoglycan From the Microbiota by Nod1 Enhances Systemic Innate Immunity. Nat Med (2010) 16:228–31. doi: 10.1038/nm.2087 PMC4497535 20081863

[28] Shi Y Zheng W Yang K Harris KG Ni K Xue L. Intratumoral Accumulation of Gut Microbiota Facilitates CD47-Based Immunotherapy via STING Signaling. J Exp Med (2020) 217:e20192282. doi: 10.1084/jem.20192282 32142585 PMC7201921

[29] Schaupp L Muth S Rogell L Kofoed-Branzk M Melchior F Lienenklaus S. Microbiota-Induced Type I Interferons Instruct a Poised Basal State of Dendritic Cells. Cell (2020) 181:1080–96.e1019. doi: 10.1016/j.cell.2020.04.022 32380006

[30] Trompette A Gollwitzer ES Yadava K Sichelstiel AK Sprenger N Ngom-Bru C. Gut Microbiota Metabolism of Dietary Fiber Influences Allergic Airway Disease and Hematopoiesis. Nat Med (2014) 20:159–66. doi: 10.1038/nm.3444 24390308

[31] Arpaia N Campbell C Fan X Dikiy S van der Veeken J deRoos P. Metabolites Produced by Commensal Bacteria Promote Peripheral Regulatory T-Cell Generation. Nature (2013) 504:451–5. doi: 10.1038/nature12726 PMC3869884 24226773

[32] Kim M Qie Y Park J Kim CH. Gut Microbial Metabolites Fuel Host Antibody Responses. Cell Host Microbe (2016) 20:202–14. doi: 10.1016/j.chom.2016.07.001 PMC4982788 27476413

[33] Mazmanian SK Liu CH Tzianabos AO Kasper DL. An Immunomodulatory Molecule of Symbiotic Bacteria Directs Maturation of the Host Immune System. Cell (2005) 122:107–18. doi: 10.1016/j.cell.2005.05.007 16009137

[34] Mager LF Burkhard R Pett N Cooke NCA Brown K Ramay H. Microbiome-Derived Inosine Modulates Response to Checkpoint Inhibitor Immunotherapy. Sci (New York NY) (2020) 369:1481–9. doi: 10.1126/science.abc3421 32792462

[35] Belkaid Y Harrison OJ. Homeostatic Immunity and the Microbiota. Immunity (2017) 46:562–76. doi: 10.1016/j.immuni.2017.04.008 PMC5604871 28423337

[36] Poore GD Kopylova E Zhu Q Carpenter C Fraraccio S Wandro S. Microbiome Analyses of Blood and Tissues Suggest Cancer Diagnostic Approach. Nature (2020) 579:567–74. doi: 10.1038/s41586-020-2095-1 PMC7500457 32214244

[37] Daillere R Vetizou M Waldschmitt N Yamazaki T Isnard C Poirier-Colame V. Enterococcus Hirae and Barnesiella Intestinihominis Facilitate Cyclophosphamide-Induced Therapeutic Immunomodulatory Effects. Immunity (2016) 45:931–43. doi: 10.1016/j.immuni.2016.09.009 27717798

[38] Viaud S Saccheri F Mignot G Yamazaki T Daillère R Hannani D. The Intestinal Microbiota Modulates the Anticancer Immune Effects of Cyclophosphamide. Sci (New York NY) (2013) 342:971–6. doi: 10.1126/science.1240537 PMC4048947 24264990

[39] Nejman D Livyatan I Fuks G Gavert N Zwang Y Geller LT. The Human Tumor Microbiome is Composed of Tumor Type-Specific Intracellular Bacteria. Sci (New York NY) (2020) 368:973–80. doi: 10.1126/science.aay9189 PMC7757858 32467386

[40] Geller LT Barzily-Rokni M Danino T Jonas OH Shental N Nejman D. Potential Role of Intratumor Bacteria in Mediating Tumor Resistance to the Chemotherapeutic Drug Gemcitabine. Sci (New York NY) (2017) 357:1156–60. doi: 10.1126/science.aah5043 PMC5727343 28912244

[41] Sivan A Corrales L Hubert N Williams JB Aquino-Michaels K Earley ZM. Commensal Bifidobacterium Promotes Antitumor Immunity and Facilitates Anti-PD-L1 Efficacy. Sci (New York NY) (2015) 350:1084–9. doi: 10.1126/science.aac4255 PMC4873287 26541606

[42] Thaiss CA Levy M Grosheva I Zheng D Soffer E Blacher E. Hyperglycemia Drives Intestinal Barrier Dysfunction and Risk for Enteric Infection. Sci (New York NY) (2018) 359:1376–83. doi: 10.1126/science.aar3318 29519916

[43] Iida N Dzutsev A Stewart CA Smith L Bouladoux N Weingarten RA. Commensal Bacteria Control Cancer Response to Therapy by Modulating the Tumor Microenvironment. Sci (New York NY) (2013) 342:967–70. doi: 10.1126/science.1240527 PMC6709532 24264989

[44] Feng M Chen JY Weissman-Tsukamoto R Volkmer JP Ho PY McKenna KM. Macrophages Eat Cancer Cells Using Their Own Calreticulin as a Guide: Roles of TLR and Btk. Proc Natl Acad Sci USA (2015) 112:2145–50. doi: 10.1073/pnas.1424907112 PMC4343163 25646432

[45] Tseng D Volkmer JP Willingham SB Contreras-Trujillo H Fathman JW Fernhoff NB. Anti-CD47 Antibody-Mediated Phagocytosis of Cancer by Macrophages Primes an Effective Antitumor T-Cell Response. Proc Natl Acad Sci USA (2013) 110:11103–8. doi: 10.1073/pnas.1305569110 PMC3703977 23690610

[46] Gil-Cruz C Perez-Shibayama C De Martin A Ronchi F van der Borght K Niederer R. Microbiota-Derived Peptide Mimics Drive Lethal Inflammatory Cardiomyopathy. Sci (New York NY) (2019) 366:881–6. doi: 10.1126/science.aav3487 31727837

[47] Ji Q Perchellet A Goverman JM. Viral Infection Triggers Central Nervous System Autoimmunity via Activation of CD8+ T Cells Expressing Dual TCRs. Nat Immunol (2010) 11:628–34. doi: 10.1038/ni.1888 PMC2900379 20526343

[48] Bradley CP Teng F Felix KM Sano T Naskar D Block KE. Segmented Filamentous Bacteria Provoke Lung Autoimmunity by Inducing Gut-Lung Axis Th17 Cells Expressing Dual TCRs. Cell Host Microbe (2017) 22:697–704.e694. doi: 10.1016/j.chom.2017.10.007 29120746 PMC5749641

[49] Balachandran VP Luksza M Zhao JN Makarov V Moral JA Remark R. Identification of Unique Neoantigen Qualities in Long-Term Survivors of Pancreatic Cancer. Nature (2017) 551:512–6. doi: 10.1038/nature24462 PMC6145146 29132146

[50] Vujanovic L Mandic M Olson WC Kirkwood JM Storkus WJ. A Mycoplasma Peptide Elicits Heteroclitic CD4+ T Cell Responses Against Tumor Antigen MAGE-A6. Clin Cancer Res (2007) 13:6796–806. doi: 10.1158/1078-0432.CCR-07-1909 18006782

[51] Vétizou M Pitt JM Daillère R Lepage P Waldschmitt N Flament C. Anticancer Immunotherapy by CTLA-4 Blockade Relies on the Gut Microbiota. Sci (New York NY) (2015) 350:1079–84. doi: 10.1126/science.aad1329 PMC4721659 26541610

[52] Bessell CA Isser A Havel JJ Lee S Bell DR Hickey JW. Commensal Bacteria Stimulate Antitumor Responses *via* T Cell Cross-Reactivity. JCI Insight (2020) 5:e135597. doi: 10.1172/jci.insight.135597 PMC7205429 32324171

[53] Fluckiger A Daillère R Sassi M Sixt BS Liu P Loos F. Cross-Reactivity Between Tumor MHC Class I-Restricted Antigens and an Enterococcal Bacteriophage. Sci (New York NY) (2020) 369:936–42. doi: 10.1126/science.aax0701 32820119

[54] Nicholson JK Holmes E Kinross J Burcelin R Gibson G Jia W. Host-Gut Microbiota Metabolic Interactions. Sci (New York NY) (2012) 336:1262–7. doi: 10.1126/science.1223813 22674330

[55] Levy M Blacher E Elinav E. Microbiome, Metabolites and Host Immunity. Curr Opin Microbiol (2017) 35:8–15. doi: 10.1016/j.mib.2016.10.003 27883933

[56] Iraporda C Errea A Romanin DE Cayet D Pereyra E Pignataro O. Lactate and Short Chain Fatty Acids Produced by Microbial Fermentation Downregulate Proinflammatory Responses in Intestinal Epithelial Cells and Myeloid Cells. Immunobiology (2015) 220:1161–9. doi: 10.1016/j.imbio.2015.06.004 26101138

[57] Vinolo MA Rodrigues HG Hatanaka E Sato FT Sampaio SC Curi R. Suppressive Effect of Short-Chain Fatty Acids on Production of Proinflammatory Mediators by Neutrophils. J Nutr Biochem (2011) 22:849–55. doi: 10.1016/j.jnutbio.2010.07.009 21167700

[58] Macia L Tan J Vieira AT Leach K Stanley D Luong S. Metabolite-Sensing Receptors GPR43 and GPR109A Facilitate Dietary Fibre-Induced Gut Homeostasis Through Regulation of the Inflammasome. Nat Commun (2015) 6:6734. doi: 10.1038/ncomms7734 25828455

[59] Chang PV Hao L Offermanns S Medzhitov R. The Microbial Metabolite Butyrate Regulates Intestinal Macrophage Function via Histone Deacetylase Inhibition. Proc Natl Acad Sci USA (2014) 111:2247–52. doi: 10.1073/pnas.1322269111 PMC3926023 24390544

[60] Gurav A Sivaprakasam S Bhutia YD Boettger T Singh N Ganapathy V. Slc5a8, a Na+-Coupled High-Affinity Transporter for Short-Chain Fatty Acids, Is a Conditional Tumour Suppressor in Colon That Protects Against Colitis and Colon Cancer Under Low-Fibre Dietary Conditions. Biochem J (2015) 469:267–78. doi: 10.1042/bj20150242 PMC4943859 25984582

[61] White CA Pone EJ Lam T Tat C Hayama KL Li G. Histone Deacetylase Inhibitors Upregulate B Cell microRNAs That Silence AID and Blimp-1 Expression for Epigenetic Modulation of Antibody and Autoantibody Responses. J Immunol (Baltimore Md: 1950) (2014) 193:5933–50. doi: 10.4049/jimmunol.1401702 PMC4258531 25392531

[62] Smith PM Howitt MR Panikov N Michaud M Gallini CA Bohlooly YM. The Microbial Metabolites, Short-Chain Fatty Acids, Regulate Colonic Treg Cell Homeostasis. Sci (New York NY) (2013) 341:569–73. doi: 10.1126/science.1241165 PMC3807819 23828891

[63] Coutzac C Jouniaux JM Paci A Schmidt J Mallardo D Seck A. Systemic Short Chain Fatty Acids Limit Antitumor Effect of CTLA-4 Blockade in Hosts With Cancer. Nat Commun (2020) 11:2168. doi: 10.1038/s41467-020-16079-x 32358520 PMC7195489

[64] Nomura M Nagatomo R Doi K Shimizu J Baba K Saito T. Association of Short-Chain Fatty Acids in the Gut Microbiome With Clinical Response to Treatment With Nivolumab or Pembrolizumab in Patients With Solid Cancer Tumors. JAMA Netw Open (2020) 3:e202895. doi: 10.1001/jamanetworkopen.2020.2895 32297948 PMC7163404

[65] Peng Z Cheng S Kou Y Wang Z Jin R Hu H. The Gut Microbiome Is Associated With Clinical Response to Anti-PD-1/PD-L1 Immunotherapy in Gastrointestinal Cancer. Cancer Immunol Res (2020) 8:1251–61. doi: 10.1158/2326-6066.CIR-19-1014 32855157

[66] Renner K Bruss C Schnell A Koehl G Becker HM Fante M. Restricting Glycolysis Preserves T Cell Effector Functions and Augments Checkpoint Therapy. Cell Rep (2019) 29:135–50.e139. doi: 10.1016/j.celrep.2019.08.068 31577944

[67] Chen J Cao X Li B Zhao Z Chen S Lai SWT. Warburg Effect Is a Cancer Immune Evasion Mechanism Against Macrophage Immunosurveillance. Front Immunol (2020) 11:621757. doi: 10.3389/fimmu.2020.621757 33603751 PMC7884830

[68] Carriche GM Almeida L Stuve P Velasquez L Dhillon-LaBrooy A Roy U. Regulating T-Cell Differentiation Through the Polyamine Spermidine. J Allergy Clin Immunol (2021) 147:335–48.e311. doi: 10.1016/j.jaci.2020.04.037 32407834

[69] He B Hoang TK Wang T Ferris M Taylor CM Tian X. Resetting Microbiota by Lactobacillus Reuteri Inhibits T Reg Deficiency-Induced Autoimmunity via Adenosine A2A Receptors. J Exp Med (2017) 214:107–23. doi: 10.1084/jem.20160961 PMC5206500 27994068

[70] Wang T Gnanaprakasam JNR Chen X Kang S Xu X Sun H. Inosine Is an Alternative Carbon Source for CD8(+)-T-Cell Function Under Glucose Restriction. Nat Metab (2020) 2:635–47. doi: 10.1038/s42255-020-0219-4 PMC7371628 32694789

[71] Chaput N Lepage P Coutzac C Soularue E Le Roux K Monot C. Baseline Gut Microbiota Predicts Clinical Response and Colitis in Metastatic Melanoma Patients Treated With Ipilimumab. Ann Oncol (2017) 28:1368–79. doi: 10.1093/annonc/mdx108 28368458

[72] Frankel AE Coughlin LA Kim J Froehlich TW Xie Y Frenkel EP. Metagenomic Shotgun Sequencing and Unbiased Metabolomic Profiling Identify Specific Human Gut Microbiota and Metabolites Associated With Immune Checkpoint Therapy Efficacy in Melanoma Patients. Neoplasia (2017) 19:848–55. doi: 10.1016/j.neo.2017.08.004 PMC5602478 28923537

[73] Gopalakrishnan V Spencer CN Nezi L Reuben A Andrews MC Karpinets TV. Gut Microbiome Modulates Response to Anti-PD-1 Immunotherapy in Melanoma Patients. Sci (New York NY) (2018) 359:97–103. doi: 10.1126/science.aan4236 PMC5827966 29097493

[74] Matson V Fessler J Bao R Chongsuwat T Zha Y Alegre ML. The Commensal Microbiome is Associated With Anti-PD-1 Efficacy in Metastatic Melanoma Patients. Sci (New York NY) (2018) 359:104–8. doi: 10.1126/science.aao3290 PMC6707353 29302014

[75] Wind TT Gacesa R Vich Vila A de Haan JJ Jalving M Weersma RK. Gut Microbial Species and Metabolic Pathways Associated With Response to Treatment With Immune Checkpoint Inhibitors in Metastatic Melanoma. Melanoma Res (2020) 30:235–46. doi: 10.1097/CMR.0000000000000656 31990790

[76] Jin Y Dong H Xia L Yang Y Zhu Y Shen Y. The Diversity of Gut Microbiome Is Associated With Favorable Responses to Anti-Programmed Death 1 Immunotherapy in Chinese Patients With NSCLC. J Thorac Oncol (2019) 14:1378–89. doi: 10.1016/j.jtho.2019.04.007 31026576

[77] Hakozaki T Richard C Okuma Y Derosa L Elkrief A Zitvogel L. Gut Microbiome to Predict Efficacy and Immune-Related Toxicities in Patients With Advanced Non-Small Cell Lung Cancer Treated With Anti-PD-1/PD-L1 Antibody-Based Immunotherapy. Am Soc Clin Oncol (2020) 38:3095. doi: 10.1200/JCO.2020.38.15_suppl.3095

[78] Katayama Y Yamada T Shimamoto T Iwasaku M Kaneko Y Uchino J. The Role of the Gut Microbiome on the Efficacy of Immune Checkpoint Inhibitors in Japanese Responder Patients With Advanced non-Small Cell Lung Cancer. Transl Lung Cancer Res (2019) 8:847–53. doi: 10.21037/tlcr.2019.10.23 PMC6976345 32010563

[79] Song P Yang D Wang H Cui X Si X Zhang X. Relationship Between Intestinal Flora Structure and Metabolite Analysis and Immunotherapy Efficacy in Chinese NSCLC Patients. Thorac Cancer (2020) 11:1621–32. doi: 10.1111/1759-7714.13442 PMC7262920 32329229

[80] Fukuoka S Daisuke M Togashi Y Sugiyama E Udagawa H Kirita K. Association of Gut Microbiome With Immune Status and Clinical Response in Solid Tumor Patients Who Received on Anti-PD-1 Therapies. Am Soc Clin Oncol (2018) 36:3011. doi: 10.1200/JCO.2018.36.15_suppl.3011

[81] Routy B Le Chatelier E Derosa L Duong CPM Alou MT Daillère R. Gut Microbiome Influences Efficacy of PD-1-Based Immunotherapy Against Epithelial Tumors. Sci (New York NY) (2018) 359:91–7. doi: 10.1126/science.aan3706 29097494

[82] Agarwal A Modliszewski J Davey L Reyes-Martinez M Runyambo D Corcoran D. Investigating the Role of the Gastrointestinal Microbiome in Response to Immune Checkpoint Inhibitors (ICIs) Among Patients (Pts) With Metastatic Renal Cell Carcinoma (mRCC). Am Soc Clin Oncol (2020) 38:730. doi: 10.1200/JCO.2020.38.6_suppl.730

[83] Heshiki Y Vazquez-Uribe R Li J Ni Y Quainoo S Imamovic L. Predictable Modulation of Cancer Treatment Outcomes by the Gut Microbiota. Microbiome (2020) 8:28. doi: 10.1186/s40168-020-00811-2 32138779 PMC7059390

[84] Yin H Yang L Peng G Yang K Mi Y Hu X. The Commensal Consortium of the Gut Microbiome Is Associated With Favorable Responses to Anti-Programmed Death Protein 1 (PD-1) Therapy in Thoracic Neoplasms. Cancer Biol Med (2021) 18. doi: 10.20892/j.issn.2095-3941.2020.0450 PMC8610161 33960176

[85] Zheng Y Wang T Tu X Huang Y Zhang H Tan D. Gut Microbiome Affects the Response to Anti-PD-1 Immunotherapy in Patients With Hepatocellular Carcinoma. J Immunother Cancer (2019) 7:193. doi: 10.1186/s40425-019-0650-9 31337439 PMC6651993

[86] Liu D Schilling B Liu D Sucker A Livingstone E Jerby-Arnon L. Integrative Molecular and Clinical Modeling of Clinical Outcomes to PD1 Blockade in Patients With Metastatic Melanoma. Nat Med (2019) 25:1916–27. doi: 10.1038/s41591-019-0654-5 PMC6898788 31792460

[87] Batten M Shanahan E Simpson R Read M Silva IP Angelatos A. Gut Microbiota Predicts Response and Toxicity With Neoadjuvant Immunotherapy. AACR (2020) 80:5734. doi: 10.1158/1538-7445.AM2020-5734

[88] Raggi D Bandini M Pederzoli F Giannatempo P Marandino L Basile G. Concomitant Antibiotics (ATBs) Use and Survival Outcomes in Patients (Pts) With Muscle-Invasive Bladder Cancer (MIBC) Treated With Neoadjuvant Pembrolizumab (PURE-01 Study). Am Soc Clin Oncol (2021) 39:449. doi: 10.1200/JCO.2021.39.6_suppl.449

[89] Tanoue T Morita S Plichta DR Skelly AN Suda W Sugiura Y. A Defined Commensal Consortium Elicits CD8 T Cells and Anti-Cancer Immunity. Nature (2019) 565:600–5. doi: 10.1038/s41586-019-0878-z 30675064

[90] Roberti MP Yonekura S Duong CPM Picard M Ferrere G Tidjani Alou M. Chemotherapy-Induced Ileal Crypt Apoptosis and the Ileal Microbiome Shape Immunosurveillance and Prognosis of Proximal Colon Cancer. Nat Med (2020) 26:919–31. doi: 10.1038/s41591-020-0882-8 32451498

[91] Ivanov II Atarashi K Manel N Brodie EL Shima T Karaoz U. Induction of Intestinal Th17 Cells by Segmented Filamentous Bacteria. Cell (2009) 139:485–98. doi: 10.1016/j.cell.2009.09.033 PMC2796826 19836068

[92] Xu X Lv J Guo F Li J Jia Y Jiang D. Gut Microbiome Influences the Efficacy of PD-1 Antibody Immunotherapy on MSS-Type Colorectal Cancer via Metabolic Pathway. Front Microbiol (2020) 11:814. doi: 10.3389/fmicb.2020.00814 32425919 PMC7212380

[93] Feng M Jiang W Kim BYS Zhang CC Fu Y-X Weissman IL. Phagocytosis Checkpoints as New Targets for Cancer Immunotherapy. Nat Rev Cancer (2019) 19:568. doi: 10.1038/s41568-019-0183-z 31462760 PMC7002027

[94] Derosa L Routy B Fidelle M Iebba V Alla L Pasolli E. Gut Bacteria Composition Drives Primary Resistance to Cancer Immunotherapy in Renal Cell Carcinoma Patients. Eur Urol (2020) 78:195–206. doi: 10.1016/j.eururo.2020.04.044 32376136

[95] Sistigu A Viaud S Chaput N Bracci L Proietti E Zitvogel L. Immunomodulatory Effects of Cyclophosphamide and Implementations for Vaccine Design. Semin Immunopathol (2011) 33:369–83. doi: 10.1007/s00281-011-0245-0 21611872

[96] Schiavoni G Sistigu A Valentini M Mattei F Sestili P Spadaro F. Cyclophosphamide Synergizes With Type I Interferons Through Systemic Dendritic Cell Reactivation and Induction of Immunogenic Tumor Apoptosis. Cancer Res (2011) 71:768–78. doi: 10.1158/0008-5472.can-10-2788 21156650

[97] Ghiringhelli F Larmonier N Schmitt E Parcellier A Cathelin D Garrido C. CD4+CD25+ Regulatory T Cells Suppress Tumor Immunity But are Sensitive to Cyclophosphamide Which Allows Immunotherapy of Established Tumors to be Curative. Eur J Immunol (2004) 34:336–44. doi: 10.1002/eji.200324181 14768038

[98] Viaud S Flament C Zoubir M Pautier P LeCesne A Ribrag V. Cyclophosphamide Induces Differentiation of Th17 Cells in Cancer Patients. Cancer Res (2011) 71:661–5. doi: 10.1158/0008-5472.can-10-1259 21148486

[99] Baruch EN Youngster I Ben-Betzalel G Ortenberg R Lahat A Katz L. Fecal Microbiota Transplant Promotes Response in Immunotherapy-Refractory Melanoma Patients. Sci (New York NY) (2020) 371:602–9. doi: 10.1126/science.abb5920 33303685

[100] Davar D Dzutsev AK McCulloch JA Rodrigues RR Chauvin J-M Morrison RM. Fecal Microbiota Transplant Overcomes Resistance to Anti-PD-1 Therapy in Melanoma Patients. Sci (New York NY) (2021) 371:595–602. doi: 10.1126/science.abf3363 PMC8097968 33542131

[101] Maleki S Lenehan J Burton J Silverman M Parvathy SN El-Hajjar M. P864 Combination of Fecal Microbiota Transplantation From Healthy Donors With Anti-PD1 Immunotherapy in Treatment-Naïve Advanced or Metastatic Melanoma Patients. BMJ Specialist J (2020) 8:A11–2. doi: 10.1136/LBA2019.17

[102] Cammarota G Ianiro G Gasbarrini A. Fecal Microbiota Transplantation for the Treatment of Clostridium Difficile Infection: A Systematic Review. J Clin Gastroenterol (2014) 48:693–702. doi: 10.1097/mcg.0000000000000046 24440934

[103] Alang N Kelly CR. Weight Gain After Fecal Microbiota Transplantation. Open Forum Infect Dis (2015) 2:ofv004. doi: 10.1093/ofid/ofv004 26034755 PMC4438885

[104] Zhernakova A Kurilshikov A Bonder MJ Tigchelaar EF Schirmer M Vatanen T. Population-Based Metagenomics Analysis Reveals Markers for Gut Microbiome Composition and Diversity. Sci (New York NY) (2016) 352:565–9. doi: 10.1126/science.aad3369 PMC5240844 27126040

[105] Falony G Joossens M Vieira-Silva S Wang J Darzi Y Faust K. Population-Level Analysis of Gut Microbiome Variation. Sci (New York NY) (2016) 352:560–4. doi: 10.1126/science.aad3503 27126039

[106] Claesson MJ Jeffery IB Conde S Power SE O’Connor EM Cusack S. Gut Microbiota Composition Correlates With Diet and Health in the Elderly. Nature (2012) 488:178–84. doi: 10.1038/nature11319 22797518

[107] Muegge BD Kuczynski J Knights D Clemente JC González A Fontana L. Diet Drives Convergence in Gut Microbiome Functions Across Mammalian Phylogeny and Within Humans. Sci (New York NY) (2011) 332:970–4. doi: 10.1126/science.1198719 PMC3303602 21596990

[108] Rajilić-Stojanović M Heilig HG Tims S Zoetendal EG de Vos WM. Long-Term Monitoring of the Human Intestinal Microbiota Composition. Environ Microbiol (2012) 15:1146–59. doi: 10.1111/1462-2920.12023 23286720

[109] Makki K Deehan EC Walter J Backhed F. The Impact of Dietary Fiber on Gut Microbiota in Host Health and Disease. Cell Host Microbe (2018) 23:705–15. doi: 10.1016/j.chom.2018.05.012 29902436

[110] Hryckowian AJ Van Treuren W Smits SA Davis NM Gardner JO Bouley DM. Microbiota-Accessible Carbohydrates Suppress Clostridium Difficile Infection in a Murine Model. Nat Microbiol (2018) 3:662–9. doi: 10.1038/s41564-018-0150-6 PMC6126909 29686297

[111] Kamada N Kim YG Sham HP Vallance BA Puente JL Martens EC. Regulated Virulence Controls the Ability of a Pathogen to Compete With the Gut Microbiota. Sci (New York NY) (2012) 336:1325–9. doi: 10.1126/science.1222195 PMC3439148 22582016

[112] Ramirez-Farias C Slezak K Fuller Z Duncan A Holtrop G Louis P. Effect of Inulin on the Human Gut Microbiota: Stimulation of Bifidobacterium Adolescentis and Faecalibacterium Prausnitzii. Br J Nutr (2009) 101:541–50. doi: 10.1017/s0007114508019880 18590586

[113] Rangan P Choi I Wei M Navarrete G Guen E Brandhorst S. Fasting-Mimicking Diet Modulates Microbiota and Promotes Intestinal Regeneration to Reduce Inflammatory Bowel Disease Pathology. Cell Rep (2019) 26:2704–19.e2706. doi: 10.1016/j.celrep.2019.02.019 30840892 PMC6528490

[114] Di Biase S Lee C Brandhorst S Manes B Buono R Cheng CW. Fasting-Mimicking Diet Reduces HO-1 to Promote T Cell-Mediated Tumor Cytotoxicity. Cancer Cell (2016) 30:136–46. doi: 10.1016/j.ccell.2016.06.005 PMC5388544 27411588

[115] Wu GD Chen J Hoffmann C Bittinger K Chen YY Keilbaugh SA. Linking Long-Term Dietary Patterns With Gut Microbial Enterotypes. Sci (New York NY) (2011) 334:105–8. doi: 10.1126/science.1208344 PMC3368382 21885731

[116] Laute-Caly DL Raftis EJ Cowie P Hennessy E Holt A Panzica DA. The Flagellin of Candidate Live Biotherapeutic Enterococcus Gallinarum MRx0518 is a Potent Immunostimulant. Sci Rep (2019) 9:801. doi: 10.1038/s41598-018-36926-8 30692549 PMC6349862

[117] Drake CG Pachynski RK Subudhi SK McNeel DG Antonarakis ES Bauer TM. Safety and Preliminary Immunogenicity of JNJ-64041809, a Live Attenuated, Double-Deleted Listeria Monocytogenes-Based Immunotherapy, in Metastatic Castration-Resistant Prostate Cancer (mCRPC). J Clin Oncol (2019) 37:e16509–9. doi: 10.1200/JCO.2019.37.15_suppl.e16509 PMC9184270 34257408

[118] Chowdhury S Castro S Coker C Hinchliffe TE Arpaia N Danino T. Programmable Bacteria Induce Durable Tumor Regression and Systemic Antitumor Immunity. Nat Med (2019) 25:1057–63. doi: 10.1038/s41591-019-0498-z PMC6688650 31270504

[119] Gurbatri CR Lia I Vincent R Coker C Castro S Treuting PM. Engineered Probiotics for Local Tumor Delivery of Checkpoint Blockade Nanobodies. Sci Trans Med (2020) 12:eaax0876. doi: 10.1126/scitranslmed.aax0876 PMC7685004 32051224

[120] Leventhal DS Sokolovska A Li N Plescia C Kolodziej SA Gallant CW. Immunotherapy With Engineered Bacteria by Targeting the STING Pathway for Anti-Tumor Immunity. Nat Commun (2020) 11:2739. doi: 10.1038/s41467-020-16602-0 32483165 PMC7264239

[121] Uribe-Herranz M Rafail S Beghi S Gil-de-Gomez L Verginadis I Bittinger K. Gut Microbiota Modulate Dendritic Cell Antigen Presentation and Radiotherapy-Induced Antitumor Immune Response. J Clin Invest (2020) 130:466–79. doi: 10.1172/JCI124332 PMC6934221 31815742

[122] Uribe-Herranz M Bittinger K Rafail S Guedan S Pierini S Tanes C. Gut Microbiota Modulates Adoptive Cell Therapy *via* CD8alpha Dendritic Cells and IL-12. JCI Insight (2018) 3:e94952. doi: 10.1172/jci.insight.94952 PMC5916241 29467322

[123] Elkrief A El Raichani L Richard C Messaoudene M Belkaid W Malo J. Antibiotics are Associated With Decreased Progression-Free Survival of Advanced Melanoma Patients Treated With Immune Checkpoint Inhibitors. Oncoimmunology (2019) 8:e1568812. doi: 10.1080/2162402X.2019.1568812 30906663 PMC6422373

[124] Derosa L Hellmann MD Spaziano M Halpenny D Fidelle M Rizvi H. Negative Association of Antibiotics on Clinical Activity of Immune Checkpoint Inhibitors in Patients With Advanced Renal Cell and non-Small-Cell Lung Cancer. Ann Oncol (2018) 29:1437–44. doi: 10.1093/annonc/mdy103 PMC6354674 29617710

[125] Zhao S Gao G Li W Li X Zhao C Jiang T. Antibiotics are Associated With Attenuated Efficacy of Anti-PD-1/PD-L1 Therapies in Chinese Patients With Advanced non-Small Cell Lung Cancer. Lung Cancer (2019) 130:10–7. doi: 10.1016/j.lungcan.2019.01.017 30885328

[126] Hakozaki T Richard C Elkrief A Hosomi Y Benlaifaoui M Mimpen I. The Gut Microbiome Associates With Immune Checkpoint Inhibition Outcomes in Patients With Advanced Non-Small Cell Lung Cancer. Cancer Immunol Res (2020) 8:1243–50. doi: 10.1158/2326-6066.CIR-20-0196 32847937

[127] Pinato DJ Howlett S Ottaviani D Urus H Patel A Mineo T. Association of Prior Antibiotic Treatment With Survival and Response to Immune Checkpoint Inhibitor Therapy in Patients With Cancer. JAMA Oncol (2019) 5:1774–8. doi: 10.1001/jamaoncol.2019.2785 PMC6743060 31513236

[128] Lalani AA Xie W Braun DA Kaymakcalan M Bosse D Steinharter JA. Effect of Antibiotic Use on Outcomes With Systemic Therapies in Metastatic Renal Cell Carcinoma. Eur Urol Oncol (2020) 3:372–81. doi: 10.1016/j.euo.2019.09.001 PMC9163676 31562048

[129] Skelly AN Sato Y Kearney S Honda K. Mining the Microbiota for Microbial and Metabolite-Based Immunotherapies. Nat Rev Immunol (2019) 19:305–23. doi: 10.1038/s41577-019-0144-5 30858494

[130] Nam GH Hong Y Choi Y Kim GB Kim YK Yang Y. An Optimized Protocol to Determine the Engulfment of Cancer Cells by Phagocytes Using Flow Cytometry and Fluorescence Microscopy. J Immunol Methods (2019) 470:27–32. doi: 10.1016/j.jim.2019.04.007 31034881

[131] Poh CM Zheng J Channappanavar R Chang ZW Nguyen THO Rénia L. Multiplex Screening Assay for Identifying Cytotoxic CD8(+) T Cell Epitopes. Front Immunol (2020) 11:400. doi: 10.3389/fimmu.2020.00400 32218786 PMC7078160

[132] Stein RR Tanoue T Szabady RL Bhattarai SK Olle B Norman JM. Computer-Guided Design of Optimal Microbial Consortia for Immune System Modulation. eLife (2018) 7:e30916. doi: 10.7554/eLife.30916 29664397 PMC5959721

[133] Marcos-Zambrano LJ Karaduzovic-Hadziabdic K Loncar Turukalo T Przymus P Trajkovik V Aasmets O. Applications of Machine Learning in Human Microbiome Studies: A Review on Feature Selection, Biomarker Identification, Disease Prediction and Treatment. Front Microbiol (2021) 12:634511. doi: 10.3389/fmicb.2021.634511 33737920 PMC7962872

[134] Maldonado-Gómez MX Martínez I Bottacini F O’Callaghan A Ventura M van Sinderen D. Stable Engraftment of Bifidobacterium Longum AH1206 in the Human Gut Depends on Individualized Features of the Resident Microbiome. Cell Host Microbe (2016) 20:515–26. doi: 10.1016/j.chom.2016.09.001 27693307

[135] Shepherd ES DeLoache WC Pruss KM Whitaker WR Sonnenburg JL. An Exclusive Metabolic Niche Enables Strain Engraftment in the Gut Microbiota. Nature (2018) 557:434–8. doi: 10.1038/s41586-018-0092-4 PMC6126907 29743671

[136] Litvak Y Byndloss MX Bäumler AJ. Colonocyte Metabolism Shapes the Gut Microbiota. Sci (New York NY) (2018) 362:eaat9076. doi: 10.1126/science.aat9076 PMC6296223 30498100

[137] Zheng D Liwinski T Elinav E. Interaction Between Microbiota and Immunity in Health and Disease. Cell Res (2020) 30:492–506. doi: 10.1038/s41422-020-0332-7 32433595 PMC7264227

